# Timing and mechanism of conceptus demise in a complement regulatory membrane protein deficient mouse

**DOI:** 10.1111/aji.12997

**Published:** 2018-06-20

**Authors:** Michael P. Triebwasser, Xiaobo Wu, Paula Bertram, Dennis E. Hourcade, Donald Michael Nelson, John P. Atkinson

**Affiliations:** ^1^ Department of Medicine Division of Rheumatology Washington University School of Medicine St. Louis MO USA; ^2^ Department of Obstetrics and Gynecology Division of Maternal‐Fetal Medicine, Ultrasound and Genetics Washington University School of Medicine St. Louis MO USA

**Keywords:** alternative pathway, C3b deposition, complement membrane regulatory activity deficiency, complement system, conceptus demise, Crry, fusion of the allantois to the chorion

## Abstract

**Problem:**

Crry is a widely expressed type 1 transmembrane complement regulatory protein in rodents which protects self‐tissue by downregulating C3 activation. *Crry* ^*−/−*^ concepti produced by *Crry* ^*+/−*^ × *Crry* ^*+/−*^ matings are attacked by maternal complement system leading to loss before day 10. The membrane attack complex is not the mediator of this death. We hypothesized that the ability of C3b to engage the alternative pathway's feedback loop relatively unchecked on placental membranes induces the lesion yielding the demise of the Crry^−/−^ mouse.

**Method of Study:**

We investigated the basis of *Crry* ^*−/−*^ conceptus demise by depleting maternal complement with cobra venom factor and blocking antibodies. We monitored their effects primarily by genotyping and histologic analyses.

**Results:**

We narrowed the critical period of the complement effect from 6.5 to 8.5 days post‐coitus (dpc), which is immediately after the conceptus is exposed to maternal blood. Deposition by 5.5 dpc of maternal C3b on the placental vasculature lacking Crry^−/−^ yielded loss of the conceptus by 8.5 dpc. Fusion of the allantois to the chorion during placental assembly did not occur, fetal vessels originating in the allantois did not infiltrate the chorioallantoic placenta, the chorionic plate failed to develop, and the labyrinthine component of the placenta did not mature.

**Conclusion:**

Our data are most consistent with the deposition of C3b being responsible for the failure of the allantois to fuse to the chorion leading to subsequent conceptus demise.

## 1 INTRODUCTION

**Figure 1:**
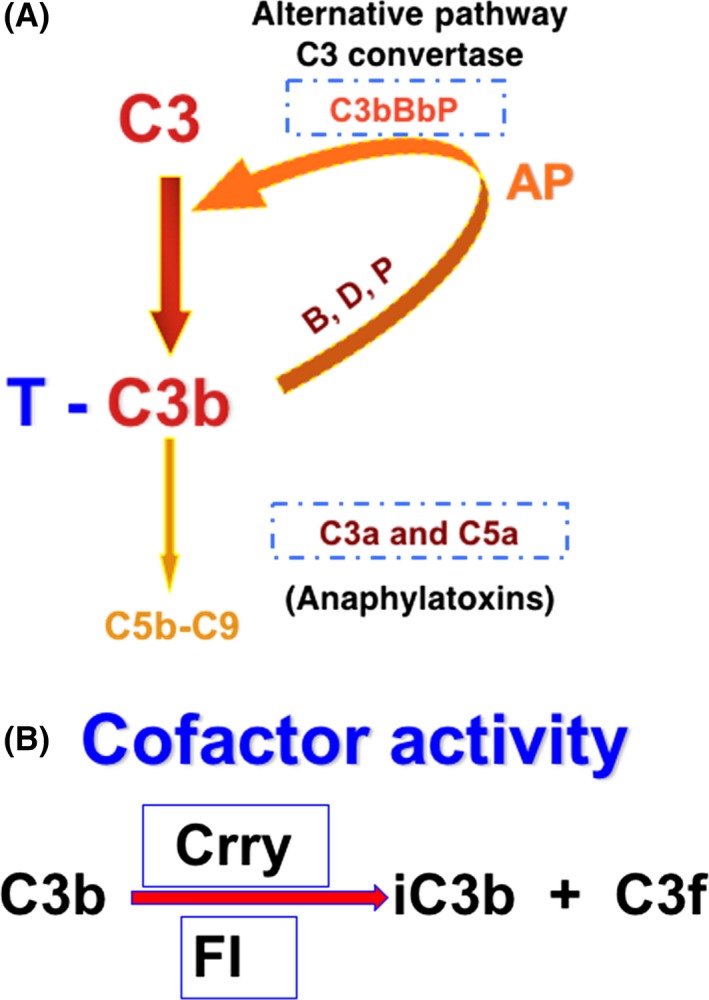
Alternative pathway of complement activation: feedback loop and regulation by cofactor activity. A, Four plasma proteins, C3, factor B (FB), factor D (FD), and properdin (P) assemble into the AP C3 convertase. C3 convertase has a continuous activity that generates a basal level of C3b. C3b engages the zymogen Factor B (FB) that is then cleaved by the serine protease Factor D (FD) to generate Bb and Ba. Bb remains bound to C3b while Ba is liberated (not shown). C3bBb is an active, albeit transient, AP enzyme complex that cleaves C3 to form C3a and C3b. C3a is a small peptide anaphylatoxin when released. C3b covalently binds to a nearby target (T) surface forming an ester linkage. Properdin binds to and stabilizes the C3bBb complex, increasing the half‐life 5‐ to 10fold. Thus, the cleavage of C3 to C3b may result in a potent positive feedback amplification loop. The AP C5 convertase (C3b)_2_ BbP (not shown) cleaves C5 to C5a and C5b. C5a is a potent anaphylatoxin. C5b binds C6 and C7 and C5b,6,7 complex attaches to a membrane. The binding of C8 followed by multiple C9s then completes the formation of the membrane attack complex (MAC), which has the capacity to perturb cellular membranes including generating pores to lyse cells. B, Crry mediates membrane cofactor activity in the mouse. After Crry binds to C3b, then Factor I (FI), a plasma serine protease, can now cleave C3b to iC3b. iC3b has no hemolytic potential because it does not engage FB to initiate the feedback loop. Crry has intrinsic complement regulatory activity in that it primarily acts on the same cell on which it is expressed^32^

The alternative pathway (AP) of the complement system is vital to host defense against pathogens yet also contributes to autoimmune and inflammatory diseases. In host defense, the effects of complement activation are largely dependent on the cleavage of C3 by the C3 convertases, enzyme complexes assembled during complement activation (Figure 1A). C3 cleavage products opsonize targets for clearance by phagocytic cells, promote inflammation, and perturb cell surfaces via the membrane attack complex (MAC; C5b‐9).

Regulation of convertase activity is required to maintain homeostasis and protect self‐tissue. Membrane cofactor protein (MCP; CD46) and decay accelerating factor (DAF; CD55) are proteins expressed on most healthy cells that regulate convertases. Factor H (FH) is an abundant plasma protein that prevents formation of and dissociates convertases via decay accelerating activity and cofactor activity (Figure 1B). Absence of these normal regulators of the AP are the cause of a number of human diseases, including atypical hemolytic uremic syndrome (aHUS),^1^, ^2^, ^3^, ^4^, ^5^, ^6^ C3 glomerulopathies (C3GN),^1^, ^2^, ^3^, ^4^, ^5^, ^6^age‐related macular degeneration (AMD),^7^, ^8^, ^9^, ^10^, ^11^ and protein‐losing enteropathy.^12^

Crry is a widely expressed type 1 transmembrane protein in rodents that downregulates the AP. It has strong cofactor activity for C3b and moderate decay accelerating activity for the classical pathway.^13^, ^14^, ^15^ Crry's broad expression profile and regulatory activities are similar to membrane cofactor protein (MCP/CD46) in primates (Figure 1B).^16^ MCP has a limited expression profile in rodents, being present primarily on the inner acrosomal membrane of spermatozoa.^9^, ^16^, ^17^, ^18^ *Crry* ^*−/−*^ concepti produced by *Crry* ^*+/−*^ × *Crry* ^*+/−*^ matings are attacked by the maternal AP leading to loss before 10 days.^15^ Notably, *Crry* ^*−/−*^ pups can be rescued if the mother is deficient in any 1 of the 4 components (C3, FB, FD, and properdin) of the AP (13, 14, 15 and X. Wu and J.P. Atkinson, unpublished). Fetal loss that occurs is not dependent on antibody (μ*MT* ^*−/−*^ background) or the classical or lectin pathway of complement (*C4* ^*−/−*^ background).^13^ Moreover, demise is not mediated by the MAC, as the C6‐deficient mouse conceptus does not rescue the demise phenotype.^19^ However, C5a may play a minor role: *C5* ^*−/−*^ background led to ~5% of offspring being *Crry* ^*−/−*^ although much less than the expected 25% of offspring.^13^ These results indicate that fetal loss occurs primarily through AP‐directed events, prior to formation of the C5 convertase and the membrane attack complex.

Herein, we test the hypothesis that the ability of C3b to engage the AP feedback loop in the absence of proper membrane regulation induces the placental lesion yielding the demise of the Crry^−/−^ mouse. Specifically, we surmise that a reduction in maternal AP activity at ~6.5 days post‐coitus (dpc) would prevent conceptus demise. The mouse blastocyst enters the uterus at 4.5 dpc yet the conceptus is not directly exposed to maternal blood until 5.0‐6.5 dpc.^20^ As early as 6.5 but completely by 7.5 dpc, the ectoplacental cone cells that will evolve into the chorioallantoic placenta are bathed in maternal blood, thereby exposing to proteins of the complement cascade. Neutrophil depletion and C3a receptor blockade failed to rescue the conceptus. Overall, the results further suggest that C3b deposition is responsible for the loss of the conceptus.^13^, ^14^, ^15^, ^19^

## 2 MATERIALS AND METHODS

### 2.1 Mouse breeding and genotyping

Mice were bred and maintained under pathogen‐free conditions at Washington University School of Medicine (WUSM) in St. Louis, MO in accordance with institutional animal care guidelines. The *Crry* knockout mouse was originally generated by Molina and colleagues^14^ and has been maintained at WUSM. The *Crry* ^*−/−*^ allele was genotyped by PCR as described.^15^ The *C3aR* knockout mouse was a gift from Richard Wetsel (University of Texas, Houston) and genotyped by PCR.

### 2.2 Timed matings and harvesting embryos

After female mice were placed in the male's cage, each subsequent day the female was checked for a vaginal plug. The day of plug observance was assigned 0.5 dpc. Mice were expected to deliver at 19.5 dpc. Pregnant mice were sacrificed by CO_2_ asphyxiation in accordance with institutional guidelines. The uterus was then removed and each implantation site was separated surgically. The muscular uterus was removed under a dissecting microscope while the implantation site was placed in cold PBS. Pregnant mice were routinely sacrificed at day 11.5 dpc. Implantation sites, including the concepti and extraembryonic membrane surrounded by decidua, were weighed to confirm the dpc. Genotyping was performed on each litter. To accomplish this, the conceptus was removed, washed 7× in cold PBS in a microtiter plate, and digested in proteinase K (20 μg/mL) overnight at 55°C. DNA was precipitated, suspended in 10 mmol/L Tris, 0.1 mmol/L EDTA, and then analyzed by PCR as described previously.^15^

### 2.3 Transcardial perfusion

Mice were anesthetized with ketamine/xylazine (ketamine from Henry Schein, Dublin, OH; xylazine from Department of Comparative Medicine, WUSM, St. Louis, MO). Transcardial perfusion with 50 mL of Dulbecco's phosphate‐buffered saline (DPBS; Sigma‐Aldrich, St. Louis, MO) containing 20 U/mL of heparin (Sigma‐Aldrich) was performed to remove plasma and red blood cells (RBCs) from the vasculature.

### 2.4 Frozen section histology

Implantation sites, harvested as above, were dehydrated in 20% sucrose overnight at 4°C, flash frozen in optimal cutting temperature (Sakura USA, Torrance, CA) with 2‐methylbutane, and cooled with dry ice. Cassettes were stored at −80°C. Frozen sections (7 μm) were prepared on a Leica CryoStat. For granulocyte‐differentiation antigen‐1 (Gr‐1) staining, frozen slides were fixed in pre‐chilled acetone at room temperature (RT). Endogenous peroxidase was quenched with 0.3% H_2_O_2_ (Sigma‐Aldrich) in methanol (Fisher Scientific, St. Louis, MO). Blocking was performed in PBS, 1% BSA, 5% mouse serum, and 5% goat serum. RB6‐8C5 (BioXCell 3.5 mg/mL, a rat monoclonal anti‐mouse Gr‐1 Ab) was used at a dilution of 1:500 in the blocking buffer. Another anti‐Gr‐1 Ab (1A8) was used at 1:500 (BioXCell). The secondary Ab was a goat anti‐rat light chain horse radish peroxidase (HRP; Jackson Immunoresearch). Staining was visualized with DAB (3, 3′‐diaminobenzidine; Vector, IMPACT DAB KIT).

### 2.5 Immunohistology

Implantation sites were fixed in 10% formalin overnight, embedded in paraffin, 8 μm sections were rehydrated, antigen retrieval was performed, non‐specific binding was blocked, and specific immunostaining was conducted as described below. In the case of Crry staining, antigen retrieval was performed in 10 mmol/L citric acid (anhydrous; Sigma‐Aldrich), 0.05% Tween‐10, pH 6.0 in a pressure cooker for 3 minutes. Blocking and staining were performed in 1% BSA, 10% donkey serum, and 5% mouse serum in PBS. Rabbit anti‐Crry (1:1000; provided by V. Michael Holers, Division of Rheumatology, University of Colorado School of Medicine, Aurora, CO) was placed in blocking buffer overnight. Donkey anti‐rabbit HRP (Jackson Immunoresearch) was used at 1:200. Staining was visualized with diaminobenzidine (DAB).

For trophoblast staining, 7.5 dpc implantation sites were collected as described above. Antigen retrieval was accomplished with 10 mmol/L Tris‐EDTA pH 9.0 for 3 minutes in a pressure cooker. Staining was accomplished with TROMA‐I (1:50 dilution of hybridoma supernatant; Developmental Studies Hybridoma Bank, University of Iowa) and goat anti‐rat light chain horseradish peroxidase (HRP). Staining was visualized with DAB. Controls employed a second Ab only.

### 2.6 FACS analysis of cells derived from 7.5 dpc implantation sites

Implantation sites were harvested as described above. Each site was cut into 12 pieces and placed in RPMI 5% fetal bovine serum (FBS). These pieces were digested in RPMI containing 5% FBS, 300 μg/mL collagenase F (Sigma‐Aldrich), 200 μg/mL collagenase L (Sigma‐Aldrich), 500 μg/mL Dispase (Gibco), and 2 U/mL DNase‐1 (Roche) at 37°C for 30‐45 minutes with a magnetic stir bar for agitation. Cells were passed over a 70 μm strainer (BD) to create a single‐cell suspension. Implantation sites were washed in DPBS, 1% FBS, 25 mmol/L EDTA. Cells were stained for FACS with anti‐CD45 (30‐F11, BD), anti‐CD11b (M1/70, BD), anti‐Gr‐1 (RB6‐8C5, BD), and rabbit anti‐Crry followed by a donkey anti‐rabbit DyLight 488 (Jackson ImmunoResearch). Blocking for FACS was carried out employing DPBS, 1% FBS, 25 mmol/L EDTA with 5% donkey serum, and 5% mouse serum. Cells were examined employing a FACScan (BD) retrofitted with a Cytek Upgrade.

### 2.7 Cobra venom factor (CVF) treatment

Cobra venom factor (Quidel, A600) was administered intraperitoneally (20 μg/mouse) with a 31G insulin syringe (Terumo). Depletion of C3 occurs in <1 hour. C3 hemolytic and antigenic activity is undetectable for up to 3‐4 days and then there is a gradual increase to normal levels over approximately a week.^21^, ^22^

### 2.8 Neutrophil depletion

Neutrophils were depleted by intraperitoneal injection (IP) injection of RB6‐8C5, a rat IgG2b mAb against Gr‐1 (Ly6G/C). A 250 μg dose of this Ab depleted neutrophils in the periphery for 5 days and a 500 μg dose depleted for 6 days. Both doses are followed by a rebound neutrophilia (approximately a doubling of pre‐depletion levels), which we were unable to overcome with an additional IP dose 4 days after the first dose. 1A8 is a second anti‐Gr‐1 rat IgG2a Ab (BioXCell). A 500 μg dose of 1A8 depleted ~50% of the neutrophils when the peripheral blood was assayed at 72 hours.

In the initial experiments, we used RB6‐8C5 that was a gift from Emil Unanue (Washington Univ. School of Medicine, Department of Pathology and Immunology, St. Louis, MO).^23^ RB6‐8C5 was also produced within the laboratory by hybridoma cells. The mAb was purified from supernatants on a protein G column and then dialyzed against PBS. RB6 was also purchased from BioXCell (West Lebanon, NH).

## 3 RESULTS

### 3.1 Timing of AP activity required for conceptus loss and depletion of the AP by CVF

*Crry* ^*−/−*^ products of conception are known to undergo demise before 10.5 dpc with maternal AP components playing a critical role.^13^, ^14^, ^15^, ^24^ We sought to determine when the lethal AP activity occurred and we chose treatments that could suspend AP activity after implantation. CVF is a C3b analog that forms a stable AP convertase with Bb (CVFBb), and unlike host AP C3 convertases, CVFBb is not susceptible to inhibitory activity of the complement regulators.^25^ Consequently, treatment with CVF rapidly depletes circulating C3 and diminishes FB, fully exhausting complement activity.^22^

We depleted AP by injecting 20 μg CVF IP into newly pregnant mice and examined Western Blots of serum for C3. This approach yielded no detectable C3 for the next 4 days, with C3 levels returning to 50% of normal by 7 days post‐injection. The fetuses were genotyped and the placenta examined at 11.5 dpc. CVF treatment administered between 3.5 and 5.5 dpc rescued *Crry* ^*−/−*^ mice, compared to controls (Table 1). Treatment at 6.5 or 7.5 dpc led to ~50% rescue. Of note, CVF treatment had no adverse effect on pup survival, as *Crry* ^*−/−*^ pups were born to CVF‐treated mothers similar to controls (Table 2), matured as expected, and *Crry* ^*−/−*^ females were fertile (not shown).

**Table 1:** Treatment with CVF prior to 8.5 dpc rescues *Crry* ^*−/−*^ implantations^a^

dpc of treatment (litters)	Full size implantations (fraction of total)	Resorbed implantations (fraction of total)	Resorbed implantations (% of total)	*Crry* ^*−/−*^ implantations rescued
3.5 (3)	26/27	1/27	4	Yes**
4.5 (2)	19/20	1/20	5	Yes**
5.5 (1)	10/10	0/10	0	Yes
6.5 (2)	13/15	2/15	13	Partial*
7.5 (3)	24/27	3/27	11	Partial**
8.5 (3)	17/25	8/25	32	No

CVF, cobra venom factor; dpc, day post‐coitus.

a Data derived from crosses between *Crry* ^*+/−*^ females and *Crry* ^*+/−*^ males. Approximately, 25% of the resulting concepti would be expected to be *Crry* ^*−/−*^ and resorbed in the absence of CVF treatment. Implantation sites were evaluated and genotyped at 11.5 dpc.

**P < .01 and *P < .05 compared with the proportion of full size implantations if not treated. The 5.5 dpc had only 1 treated litter; a statistical test was not performed. Partial rescue indicates the number of resorbed implantations was between a full rescue and no rescue.

**Table 2:** Treatment with CVF enables the birth of *Crry* ^*−/−*^ pups^a^

dpc of treatment (litters)	*Crry* ^*−/−*^ pups^b^ (fraction of total)	*Crry* ^*−/−*^ pups (% of total)	*Crry* ^*−/−*^pups rescued
3.5 (3)	7/17	41	Yes***
4.5 (2)	10/16	63	Yes***
5.5 (5)	14/26	53	Yes***

CVF, cobra venom factor; dpc, day post‐coitus.

a Data derived from crosses between *Crry* ^*+/−*^ females and *Crry* ^*−/−*^ males. Approximately, 50% of the concepti are expected to be *Crry* ^*−/−*^. Pups were genotyped at birth. No Crry^−/−^ pups were born when CVF treatment was omitted (7 litters).

b *Crry* ^*−/−*^ pups were normal in size and developed comparably to wild‐type mice. The *Crry* ^*−/−*^ females were fertile and delivered normal litters when crossed to *Crry* ^*+/+*^ males.

***P ≤ .005 compared with the expected percentage (0%) of *Crry* ^*−/−*^ pups in the absence of CVF treatment.

### 3.2 Effects of depletion of properdin by anti‐properdin antibody

Properdin (P) is critical for AP activity in many systems as this positive regulator stabilizes the C3 convertase 5‐ to 10 fold.^26^, ^27^, ^28^ The *P* ^*−/−*^ background rescues *Crry* ^−/−^ viability,^24^ as does *FB* ^−/−,^ 13 *FD* ^*−/−*^ (X. Wu and J.P. Atkinson, unpublished) and *C3* ^−/−.^ 14 A treatment of newly pregnant mice with a rabbit polyclonal Ab to mouse properdin also restored *Crry* ^*−/−*^ viability (Table 3).^29^

**Table 3:** Transient inhibition of properdin rescues *Crry* ^*−/−*^ implantations^a^

dpc of treatment (litters)	Full size implantations (fraction of total)	Resorbed implantations (fraction of total)	Resorbed implantations (% of total)	*Crry* ^*−/−*^ implantations rescued
3.5 + 7.5 (2)^b^	15/17	2/17	12	Yes***
6.5 + 7.5 (3)^b^	20/24	4/24	17	Yes***
6.5 + 7.5 (2)^c^	12/17	5/17	29	No**
7.5 (3)	14/29	15/29	52	No

dpc, day post‐coitus.

a Data derived from crosses between *Crry* ^*+/−*^ females and *Crry* ^*−/−*^ males. Approximately, 50% of the resulting concepti would be expected to be *Crry* ^*−/−*^ and resorbed in the absence of anti‐properdin treatment. Implantation sites were evaluated and genotyped at 11.5 dpc.

b One milligram rabbit anti‐properdin on both days.

c Five hundred microgram rabbit anti‐properdin on both days.

***P < .005 and **P < .05 compared with the proportion of full size implantations if not treated.

Treatment with a neutralizing anti‐mouse properdin mAb also rescued *Crry* ^*−/−*^ concepti.^29^ Specifically, treatment with 1 mg of the H4 hamster anti‐mouse properdin mAb at 6.5 and 7.5 dpc resulted in 32% *Crry* ^*−/−*^ pup viability, compared to the expected 0% without treatment (*P* = .0005, 25 pups, 4 litters) and the 50% viability expected from *Crry* ^*+/−*^ female × *Crry* ^*−/−*^ male.

### 3.3 Histology of the implantation site

**Figure 2:**
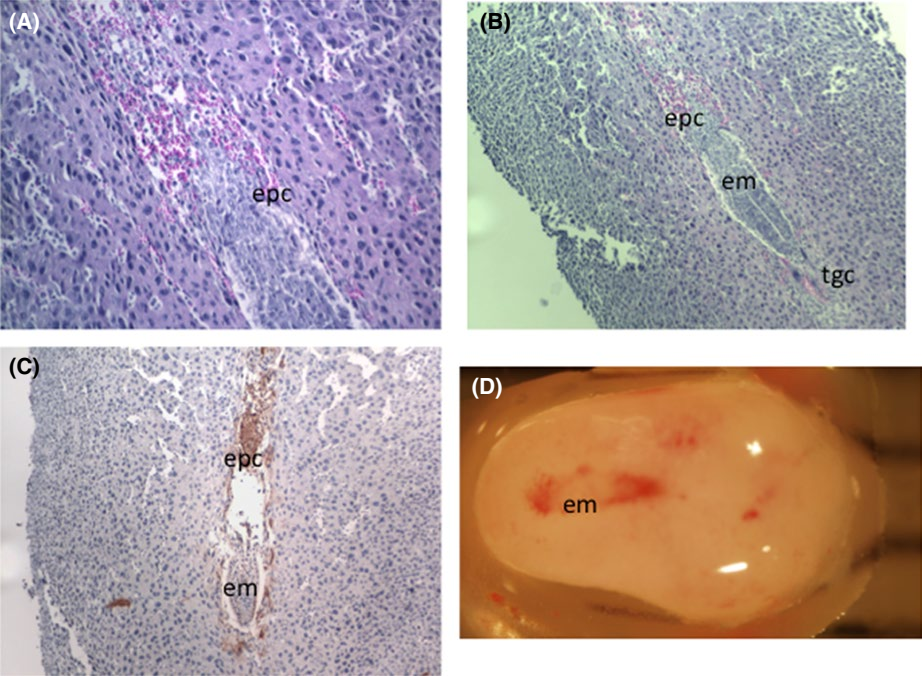
Maternal blood bathes the ectoplacental cone trophoblast at 7.5 dpc. A, Maternal red blood cells (RBCs) bathe the ectoplacental cone (epc). B, RBCs are visible at both ends of the amnionic sac. C, Among the epc are trophoblast cells that immunostain (brown) for cytokeratin 8 (TROMA‐I). D, Gross dissection of 7.5 dpc implantation site shows blood at both ends. All panels are from a 7.5 dpc litter, sacrificed without perfusion to retain maternal blood. Histology is from formalin‐fixed, paraffin‐embedded sections. A,B, are hematoxylin and eosin stained. C, Immunocytochemical staining by HRP‐conjugated anti‐cytokeratin 8. em, embryo; epc, ectoplacental cone; tgc, trophoblast giant cell. All images are 200×

**Figure 3:**
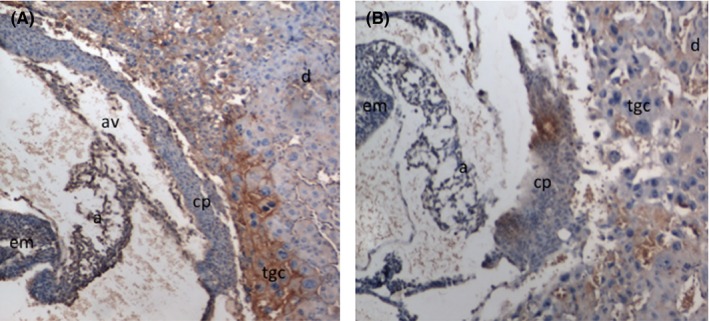
Crry^−/−^ embryos die at 8.5 dpc due to a failure of the allantoic vessels to attach to the chorionic plate. According to the Theiler classification,^19^ the allantois contacts the chorion between 7.5 and 8.75 dpc. A, The allantois of *Crry* ^*+/−*^ embryos attaches to the ectoplacental cone at 8.5 dpc and expands the chorionic plate. Notably, *Crry* ^*+/−*^ trophoblast giant cells immunostain positive (brown) for Crry. B, The allantois of *Crry* ^*−/−*^ embryos, however, does not attach properly to the ectoplacental cone at 8.5 dpc and the allantoic vessels and the trophoblast fail to develop normally. *Crry* ^*−/−*^ trophoblast giant cells do not stain positive (brown) for the Crry protein. Areas of Crry positive cells in the decidua are not of fetal origin but are maternal blood cells within maternal vessels. All sections are formalin‐fixed paraffin‐embedded sections. a, allantois; av, allantoic vessels; cp, chorionic plate; d, decidua; em, embryo; tgc, trophoblast giant cell

**Figure 4:**
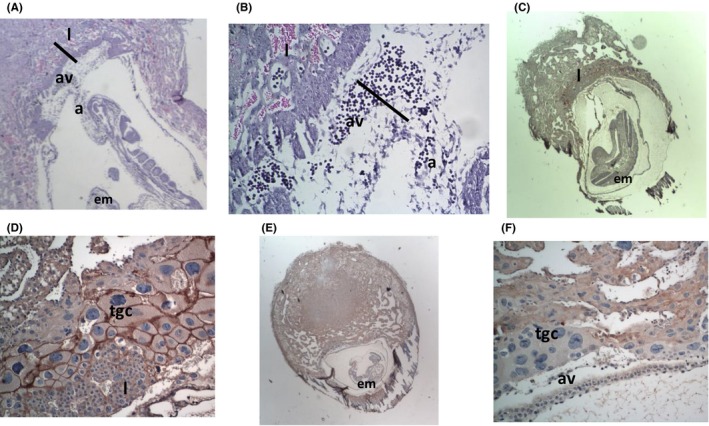
Embryos at 9.5 dpc fail to develop allantoic vessels and labyrinth. A, *Crry* ^*+/+*^ embryo is fully developed at 9.5 dpc. Its allantois has fused with the mesoderm overlaying the ectoplacental cone. Black bar delineates the labyrinth. B, The allantoic vessels (black bar) have expanded and nucleated fetal red blood cells (RBCs) are visible passing within the vessels into the labyrinth where the trophoblast interfaces with maternal RBCs without nuclei (200× of box in A). C, *Crry* ^*+/+*^ embryo at 9.5 dpc (20×). D, Trophoblast giant cells strongly express Crry (brown, rabbit anti‐Crry) and separate the maternal decidua from the labyrinth. E, *Crry* ^*−/−*^ embryo at 9.5 dpc has failed to develop. F, The *Crry* ^*−/−*^ trophoblast giant cells do not stain for Crry, the labyrinth has not developed and allantoic vessels have not expand at 9.5 dpc. av, allantoic vessels; L, labyrinth; em, embryo; tgc, trophoblast giant cell

The above results showed that conception loss was prevented if AP inhibition began before 6.5 dpc, and a partial effect existed if inhibition began by 6.5‐7.5 dpc. The ectoplacental cone cells of the mouse are precursors to the labyrinthine trophoblast in the placenta. The cone cells contact maternal RBCs as early as 5.5 dpc and are bathed in blood at 6.5 dpc.^20^ The yolk sac placenta offers nutrition to the conceptus until 9.5 dpc, when the labyrinthine placenta assumes the major role in maternal‐fetal exchange (Figure 2). Importantly, we observed that at 8.5 dpc there was a substantial difference in the size of the allantoic vessels, a lack of proliferation of the labyrinthine trophoblasts, and smaller than control embryos in *Crry* ^*−/−*^ gestations (Figure 3). Moreover, the *Crry* ^*−/−*^ implantations at 9.5 dpc failed to evolve like controls, with an undeveloped labyrinth and unexpanded chorioallantoic vessels (Figure 4).

### 3.4 Anaphylatoxin C3a and its receptor are not required for *Crry* ^*−/−*^ conceptus loss

A role for C3a in conception loss has not been studied. We tested whether C3a:C3aR signaling directed the demise of the *Crry* ^*−/−*^. C3a receptor has been shown to be important in experimental lupus nephritis.^29^ We mated *Crry* ^*+/−*^ *C3aR* ^*−/−*^ × *Crry* ^*+/−*^ *C3aR* ^*−/−*^ mice hypothesizing that deficiency of C3aR would rescue the *Crry* ^*−/−*^ embryos. The preceding cross was predicted to have 25% *Crry* ^*−/−*^ pups among the offspring, however, among the 59 viable pups, we observed no *Crry* ^*−/−*^ pups, indicating that the absence of C3aR was not sufficient to rescue *Crry* ^*−/−*^ concepti (Table 4).

**Table 4:** The *C3aR* ^*−/−*^ background does not rescue *Crry* ^*−/−*^ pups^a^

*Crry* ^*+/+*^ *C3aR* ^*−/−*^ pups (fraction of total)	*Crry* ^*+/−*^ *C3aR* ^*−/−*^ pups (fraction of total)	*Crry* ^*−/−*^ *C3aR* ^*−/−*^ pups (% of total)	Total pups	*Crry* ^*−/−*^ pups rescued
16/59	43/59	0/59	59	None

*C3aR* ^*−/−*^ *,* C3a receptor knockout mouse; dpc, day post‐coitus.

a Data derived from crosses between *Crry* ^*+/−*^ *C3aR* ^*−/−*^ females and *Crry* ^*+/−*^ *C3aR* ^*−/−*^ males. Pups were genotyped at birth. Approximately 25% of the concepti are expected to be *Crry* ^*+/+*^, 50% *Crry* ^*+/−*^, and 25% *Crry* ^*−/−*^. Loss of all *Crry* ^*−/−*^ concepti leads to approximately 66% *Crry* ^*+/−*^ and 33% *Crry* ^*+/+*^ concepti. There was no statistical difference between what was observed with these crosses and 11 *Crry* ^*+/−*^ *C3aR* ^*+/+*^ × *Crry* ^*+/−*^ *C3aR* ^*+/+*^ control litters (*P* > .05).

### 3.5 Neutrophils are not required for Crry‐/‐ conceptus loss

**Figure 5:**
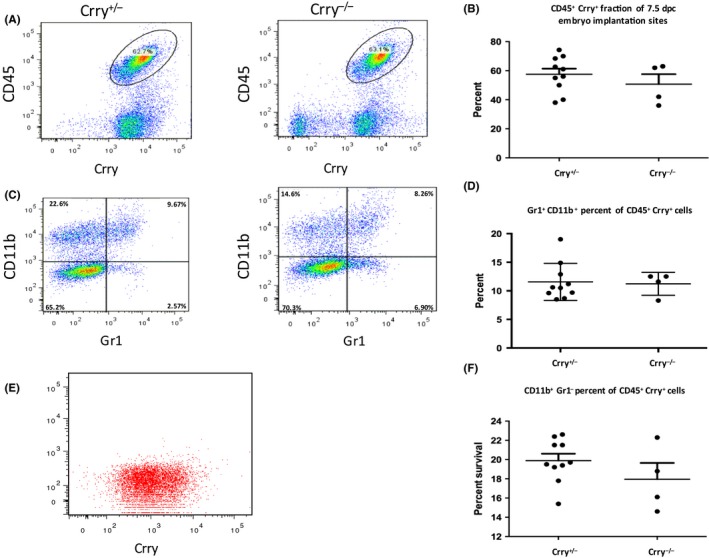
Neutrophils are present around all embryos. A, *Crry* ^*−/−*^ embryos (left panel) have 3 cell populations when stained with anti‐Crry and anti‐CD45 Abs. Crry^+^ CD45^+^ population (maternal hematopoietic‐derived cells), Crry^+^ CD45^−^ population (maternal decidua‐derived cells), and Crry^−^ CD45^−^ population (embryo‐ and trophoblast‐derived cells). *Crry* ^*+/−*^ embryos (right panel) lack the Crry^−^ CD45^−^ population; the embryo and trophoblast‐derived cells cluster with maternal decidua‐derived cells. B, There is no trend towards different proportions of hematopoietic derived (CD45^+^) cells around *Crry* ^*+/−*^ versus *Crry* ^*−/−*^ embryos. C, The Crry^+^ CD45^+^ population contains Gr‐1^+^ CD11b^+^ positive cells, neutrophils. D, There is no difference in the proportion of neutrophils dependent upon genotype. E, Staining control for anti‐Crry. *Crry* ^*−/−*^ splenocytes in red and *Crry* ^*+/−*^ in blue. F, There is no difference in the proportion of CD11b^+^ Gr‐1^−^ cells dependent upon genotype (subset of Crry^+^ CD45^+^)

In the APLS model of embryonic lethality,^30^ complement‐mediated damage is dependent on neutrophils. We observed neutrophils surrounding *Crry* ^−/−^ implantation sites on 7.5 dpc, but the impact on conception viability has not been established.^13^ We pursued this issue and first confirmed, by both FACS and immunohistochemistry, that neutrophils surrounded *Crry* ^*−/−*^ and *Crry* ^*+/−*^ implantation sites at 7.5 dpc (Figure 5). Notably, we observed no correlation between genotype and the number of neutrophils present. Also, there was no correlation between CD45^+^ as a marker of hematopoietic‐derived cells or CD11b^+^ Gr‐1^−^ cells in either *Crry* ^*−/−*^ or *Crry* ^*+/−*^ genotypes at 7.5 dpc. Neutrophils were present at implantation sites of all tested strains.

**Figure 6:**
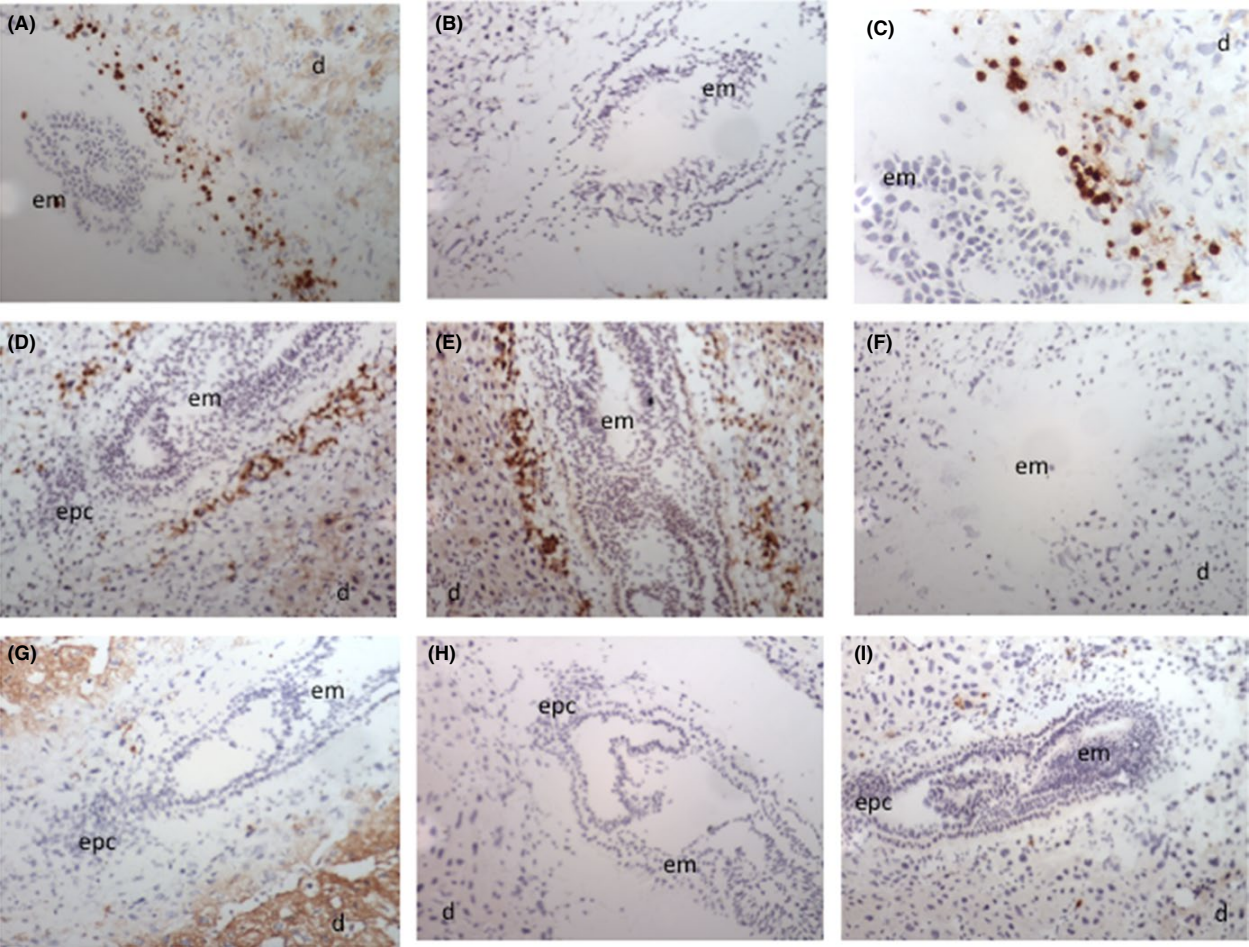
Neutrophils are present around 7.5 dpc embryos and RB6‐8C5 depletes them from around embryos. A, Neutrophils are present around the embryo at 7.5 dpc (anti‐Gr‐1 staining; 200×). B, This staining is specific for Gr‐1^+^ and is not present in the isotype control. C, At higher magnification (400×), the nuclear pattern characteristic of neutrophils can be seen inside of Gr‐1^+^ cells. D,E, Identical patterns are observed in staining for Gr‐1^+^ (RB6‐8C5, D) and Ly6G (1A8, E). F, Ly6G staining is specific to 1A8 and not seen in the isotype control. G‐I, Neutrophil depletion with 500 μg RB6‐8C5 at 4.5 dpc leads to absence of neutrophils in the tissue at 7.5 dpc of Gr‐1^+^ (G) and Ly6G^+^ cells (H,I). em, embryo; epc, ectoplacental cone

We next treated pregnant mice at 4.5 dpc with RB6‐8C5, a mAb that depletes Gr‐1 (Ly‐6C/G) positive cells, the majority of which are neutrophils, and we confirmed the absence of neutrophils at the sites of implantation at 7.5 dpc (Figure 6). Examination of the surviving concepti at 10.5 and 13.5 dpc (Table 5), along with genotyping of litters (Table 6), indicated that neutrophil depletion by RB6‐8C5 did not improve *Crry* ^*−/−*^ viability. Similarly, depletion of neutrophils by treatment of pregnant mice with an anti‐Ly6G‐specific Ab (1A8, 500 μg/mouse) had no detectable effect (Table 6). We concluded that neutrophils are not essential for loss of the *Crry* ^*−/−*^ embryos.

**Table 5:** Neutrophil depletion does not rescue *Crry* ^*−/−*^ implantations

Female × male	dpc of treatment^a^(litters)	Full size implantations (fraction of total)	Resorbed implantations (fraction of total)	Resorbed implantations (% of total)	*Crry* ^*−/−*^ implantations rescued^a^
*Crry* ^*+/−*^ * *× *Crry* ^*+/−*^ (25% Crry^−/−^ expected)	RB6/5.5 (3)	19/27	8/27	30	No
	RB6/6.5 (2)	13/17	4/17	24	No
	PBS/6.5 (3)	17/25	8/25	32	No
*Crry* ^*+/−*^ * *× *Crry* ^*−/−*^ (50% *Crry* ^*−/−*^ expected)	RB6/4.5 (2)	6/14	8/14	57	No
	RB6/4.5, 6.5 (2)	9/15	6/15	40	No
	1A8/5.5 (3)	13/21	8/21	46	No

PBS, phosphate‐buffered saline; dpc, day post‐coitus.

RB6‐8C5 (mAb anti‐Gr1) 250 μg IP in *Crry* ^*+/−*^ × *Crry* ^*+/−*^ matings and 500 μg IP in *Crry* ^*+/−*^ × *Crry* ^*−/−*^ matings. 1A8 (mAb anti‐Ly6G) 500 μg IP. Two dates listed for dpc indicates that the dose was given twice.

a No treatment condition significantly differed from the expected rate of resorptions (*P* > .05). Implantation sites were examined and genotyped at 11.5 dpc.

**Table 6:** Neutrophil depletion does not rescue *Crry* ^*−/−*^ pups^a^

dpc of treatment^b^	*Crry* ^*+/−*^ pups (fraction of total)	*Crry* ^*−/−*^ pups (fraction of total)	*Crry* ^*−/−*^ pups rescued
RB6/3.5	6/6	0/6	No
1A8/4.5	4/4	0/4	No
1A8/5.5	4/4	0/4	No

dpc, day post‐coitus.

Data derived from crosses between *Crry* ^*+/−*^ females and *Crry* ^*−/−*^ males (1 litter at each dpc of treatment). Approximately, 50% of the resulting concepti would be expected to be *Crry* ^*−/−*^. Pups were genotyped at birth.

a RB6‐8C5 (mAb anti‐Gr1), 500 μg IP; 1A8 (mAb anti‐Ly6G), 500 μg IP.

## 4 DISCUSSION

The AP of the complement system is a constant sentinel, activating continuously on surfaces and in the fluid phase.^15^, ^31^ Regulators on cell membranes and in plasma are essential to control the level of activation. Crry is widely expressed on the surface of mouse cells and carries cofactor activity.^32^ In plasma and on cellular debris, factor H (FH) performs a similar role. Crry is able via CA to permanently stop AP activation on cell membranes.^33^ In the *Crry* ^*−/−*^ mouse model, excessive AP activation leads to conceptus demise by 8.5 dpc of development, highlighting the critical role played by regulators of the AP. *Crry* ^*−/−*^ pups can be rescued by down modulating the levels of the AP activating proteins in the mother.^15^

The data herein show that transient depletion of the AP, either with CVF or an anti‐properdin Ab, is sufficient to rescue *Crry* ^*−/−*^ concepti. There is a critical window at 6.5‐7.5 dpc when AP activation leads to conceptus loss. In the case of CVF, treatment prior to this window produces full rescue, but treatment during this 24‐hour period rescues about one‐half of the concepti. Interestingly, if CVF is given at 3.5 dpc prior to implantation of the blastocyst into the decidua, maternal C3 levels rise to ~50% by 7.5 dpc (4 d after CVF treatment) but *Crry* ^*−/−*^ embryos survive. This replicates the finding that full blockade of the AP is not required for embryo survival, as haploinsufficiency of AP activating components will also rescue the concepti.^15^

### 4.1 Timing of C3 deposition

C3 deposition on the ectoplacental cone of the *Crry* ^*−/−*^ mouse occurs at approximately 7.5 dpc.^13^, ^14^ We propose this takes place because there is no membrane‐based complement inhibitor on these cells. Decay‐accelerating factor (DAF), a GPI‐anchored regulator, is not expressed in the labyrinthine placenta until after ~10.5 dpc.^13^ Other C3 regulators of complement such as FH are present in maternal blood but they are unable to compensate for Crry deficiency. For example, FH is normal in *Crry* ^*−/−*^ mouse but this complement regulator is insufficient to limit the diffuse AP activation observed on cells in *the Crry* ^*−/−*^ mouse.^15^ We surmise that the underlying cause of conceptus loss in this model is a dysregulated AP of complement activation on cell membranes.

### 4.2 Mechanism of loss

In the *Crry* ^*−/−*^ mouse model, conceptus lethality is the result of insufficient complement regulatory capacity. The current study was designed to identify the early events that contribute to the pathogenesis in this system. Previous work employing knockout mice showed that fetal loss requires maternal AP components FB, FD, properdin, and C3, that is, all 4 components of the AP. Here, we extend these studies to demonstrate that depletion of maternal AP components by 6.5 dpc, utilizing CVF treatment or neutralization of properdin with an Ab, rescues the *Crry* ^*−/−*^ embryo. Within the inherent 0.5 dpc in the Theiler staging system, the ~6.5 dpc critical time period is when extraembryonic ectoplacental cone cells become bathed in maternal blood and, consequently, immersed in maternal complement.^20^ Wild‐type components of the developing labyrinthine placentas connect to the maternal vasculature by 8.5 dpc (Figures 2 and 3), and by 9.5 dpc the labyrinthine hemochorial placenta replaces the yolk sac placenta to assume the major duty of supplying nutrients to the fetus. In the *Crry* ^*−/−*^ embryo, the fusion of the allantois to the chorion during labyrinthine assembly does not occur because the chorionic plate fails to develop and the labyrinthine trophoblast does not mature to be permeated by allantoic fetal vessels (Figures 2 and 3).

Previous investigations of placental maldevelopment in the Crry^−/−^ model have ruled out involvement of Ab,^14^ major contributions of C4 and C6.^13^, ^14^, ^15^, ^19^ We now show there is no role for neutrophils. Notably, maternal C5 deficiency has a limited restorative effect, suggesting a minor role for C5a.^13^, ^14^, ^15^, ^19^ Thus, the *Crry* ^*−/−*^ model studies confirm a cause and effect relationship between complement regulatory deficiency leading to excess AP activity and aberrant placental development. Importantly, other mouse models demonstrate that placental insufficiency can occur through different complement‐dependent scenarios: in the APS model, human autoantibodies bind autoantigens in the mouse placenta where they activate complement^34^ while in the abortion‐prone mating combination of CBA/J female × DBA/2 male complement is activated via the lectin pathway.^35^

The strengths of our study include the approaches that allowed us to identify that a deficiency of maternal C3aR or a lack of neutrophils does not prevent conceptus loss. A weakness is that we can only speculate that C3 deposition begins based on when maternal blood is apparent and when complete rescue of Crry^−/−^ concepti occurred in our CVF experiments. Our studies combined with the above discussion suggest that the detrimental effects of AP activity on the development of extraembryonic cells destined to form the labyrinthine placenta are mediated primarily by C3b and/or its derivatives. We have previously extensively assessed the timing of C3b deposition on placental tissue in the normal and complement regulatory protein (Crry) deficient mice.^14^, ^15^

### 4.3 Could dysregulation of the AP of complement yield some forms of the preeclamptic phenotype?

Preeclampsia (PE) is a syndrome that associates with placental maldevelopment in the first trimester, yielding placental dysfunction in the second half of pregnancy.^36^, ^37^ The placenta is deemed a root cause for many of the variable phenotypes of PE and immune‐based maternal‐fetal incompatibilities contribute to some forms of the PE syndrome.^38^ Notably, patients with this syndrome show greater systemic complement AP activity compared to normotensive women, but debate persists as to a cause‐effect relationship of this finding with the pathogenesis of the disorder. We recently presented studies of cohorts of autoimmune and non‐autoimmune PE patients that demonstrate an association of PE with dysfunctional complement regulatory genes, including *FI*,* FH,* and *MCP*.^39^, ^40^ These findings suggest that some cases of PE may be initiated by insufficient regulatory capacity and excessive AP complement activity during development, predisposing the placenta to maldevelopment, injury, or both during gestation. Further investigation is needed to determine the scope of excessive complement activity in human placental dysfunction.
